# Epac1 protects the retina against ischemia/reperfusion-induced neuronal and vascular damage

**DOI:** 10.1371/journal.pone.0204346

**Published:** 2018-09-20

**Authors:** Li Liu, Youde Jiang, Jena J. Steinle

**Affiliations:** Department of Ophthalmology, Visual and Anatomical Sciences, Wayne State University School of Medicine, Detroit MI, United States of America; Medical College of Wisconsin, UNITED STATES

## Abstract

We had previously reported that exchange protein for cAMP 1 (Epac1) reduced inflammatory mediators in the retina of mice and in retinal endothelial cells (REC). Since ischemia can induce retinal damage potentially through activation of inflammatory cascades, we hypothesized that Epac1 would protect the retina against neuronal and vascular damage after exposure to ischemia/reperfusion (I/R). We used Epac1 floxed and endothelial cell specific Epac1 knockout mice for this work. We exposed them to ischemia for 90 minutes followed by reperfusion. One day after I/R, some mice were used for fluorescein angiography imaging or Evan’s blue measurements of permeability. Mice were sacrificed at 2 days for neuronal measurements and at 10 days for measurements of degenerate capillaries. Data show increased leakage in the Epac1 Cre-Lox (Epac1 EC-KO) mice exposed to I/R when compared to Epac1 floxed mice with the same treatment. I/R also increased numbers of degenerate capillaries and cell loss in all retinal layers of Epac1 EC-KO mice. Retinal thickness was reduced more significantly in the Epac1 EC-KO mice compared to Epac1 floxed mice after I/R. Taken together, the data suggest that Epac1 is protective against both neuronal and vascular damage to the retina after exposure to I/R.

## Introduction

Rates of diabetes are reaching epidemic levels. In addition to diabetes, other retinal diseases are highly clinically relevant, as the loss of sight is one feared most by patients. One animal model used to mimic retinal ischemia damage is ischemia/reperfusion [1–3]. Studies have reported that toll-like receptor 4 (TLR4) contributes to retinal I/R injury [4, 5]. TLR4 was also responsible for cardiac damage in response to I/R [6]. In addition to TLR4, others reported that high mobility group box 1 (HMGB1) mediates retinal injury in response to I/R [7]. Work has shown that inhibition of HMGB1 by glycyrrhizin blocked I/R injury to the brain and retina [8].

We have shown that topical application of a β-adrenergic receptor agonist, Compound 49b, was effective in reducing diabetes-induced retinal damage [9], as well as retinal damage in response to ischemia/reperfusion (I/R) [10]. Since Compound 49b had to be delivered topically, we wanted to investigate downstream pathways that may also protect the retina. We focused on exchange protein of cAMP 1 (Epac1). We have reported that Epac1 can reduce both TLR4 and HMGB1 in retinal endothelial cells (REC)[11]. We also showed that Epac1 reduced TNFα and IL-1β levels in mouse retina and in REC grown in high glucose, and this response was Epac1-specific [12].

Since we have data that Epac1 can reduce inflammatory mediators, we wanted to investigate whether loss of Epac1 in the vasculature increased retinal damage in response to ischemia/reperfusion (I/R) injury.

## Materials and methods

### Mice

Epac1 floxed mice (B6;129S2-Rapgef3^tm1Geno/J^ mice) and B6 FVB-Tg (cdh5-cre)7Mlia/J Cre mice were purchased from Jackson Laboratories. After 2 generations, Epac1 floxed mice were bred with cdh5-Cre mice to generate conditional knockout mice in which Epac1 is eliminated in endothelial cells. We have previously reported studies using these mice. In our previous studies, we showed successful loss of Epac1 in the Epac1 EC-KO mice using genotyping, Western blotting of whole retinal lysates, and immunostaining of 10um retinal sections. The retinal sections were stained with isolectin B4 (endothelial cells) and Epac1 (red) to show little co-localization of Isolectin GS-IB4 and Epac1 in the Epac1 EC-KO mice in blood vessels (Fig 2 of previous work [12]). Mice of both sexes at 2 months of age were used for these studies. All work was reviewed and approved by the Institutional Animal Care and Use Committee of Wayne State University School of Medicine (Protocol #17-07-301) and conforms to NIH guidelines.

### Genotyping

Genomic DNA extracted from ear punch samples from 2-week-old mice was digested using one step tail DNA extraction buffer (100mM Tris, 5mM EDTA, 200mM NaCl, 1% Triton) plus proteinase K (10mg/ml) at 55°C overnight, followed by enzyme heat-inactivation at 85°C for 45 min. Sequences of primer pairs used to screen the Epac1 conditional knock out mice were as follows: Epac1: 5’->3’ mutant forward: ATT TGT CAC GTC CTG CAC GAC G, wild type forward: CTG GCC TCT CCT GAA TCT TG, common: CCT CGC TGT TGG TAA GTG GT. Cdh5-cre forward: AGG CAG CTC ACA AAG GAA CAA T; reverse: TCG TTG CAT CGA CCG GTA A; Cdh5-cre internal positive control forward: CTA GGC CAC AGA ATT GAA AGA TCT; reverse: GTA GGT GGA AAT TCT AGC ATC ATC C. KAPA2G HotStart Genotyping PCR Mix (KK5621, KAPA Biosystems) was used for standard PCR reactions with following temperatures and times: denature: 95°C 3 min, 35 cycles at 95°C, 15 sec, 60°C 15 sec, 72°C sec/kb, with final extension at 72°C 1 min.

### Ischemia/Reperfusion (I/R)

Animals were anesthetized with an intraperitoneal injection of ketamine and xylazine. Once animals were sufficiently anesthetized, a 32-gauge needle attached to an infusion line of sterile saline was used to cannulate the anterior chamber of the eye. Hydrostatic pressure of 80-90mmHg (TonoPen, Medtronic, Jacksonville, FL) was maintained for 90 minutes to induce retinal ischemia, evidenced by blanching of the iris and loss of red reflex [1, 10]. After 90 minutes, the needle was withdrawn and intraocular pressure normalized. The contralateral eye served as an intra-animal control.

### Fluorescein angiography

Retinal vascular leakage analysis was done on Epac1 floxed and Epac1 EC-KO mice. The right eye of each mouse was exposed to the I/R lesion as described above. Twenty-four hours after I/R, the pupil of the mice was dilated with tropicamide ophthalmic solution. Fifteen minutes later, mice were anesthetized by ketamine and xylazine. After mice were deeply anesthetized, 150ul of AK-FLUOR (1% W/V, AKORN, INC, Lake Forest, IL) was injected intraperitoneally. Retinal vessel leakage was photographed using a Micron IV (Phoenix Research Labs, Pleasanton, CA). Images were obtained less than 5 minutes after injection of the dye.

In an additional 8 mice (4 Epac1 floxed and 4 Epac1 EC-KO), ischemia/reperfusion was done as described above. Twenty-four hours after I/R, mice were given 200ul Evans blue (0.5% in saline, Sigma Aldrich) via the tail vein. Forty-five minutes after infusion, mice were euthanized with CO2. The retinas were carefully removed, placed into 100ul formamide, and incubated for 48hours at 55°C. After incubation, the tubes were centrifuged and transferred to a 96 well plate. The absorbance of the retina was measured at 610 [13].

### Neuronal analyses

Two days after I/R exposure, a subset of each group of mice was sacrificed for measurements of neuronal thickness, as we have done previously with the exception of staining with hematoxylin and eosin instead of toluidine blue [14]. Ten micrometer sections were taken from throughout the retina. Analyses of retinal thickness and cell numbers for each retinal layer were counted [9, 14].

### Vascular analyses

Ten days after I/R exposure, additional mice were sacrificed to measure degenerate capillaries, as we have done previously [10, 15].

### Statistics

A one-way ANOVA with Student Newman Keul’s post-hoc test was used for animal data. P<0.05 was considered statistically significant.

## Results

**Fig 1:**
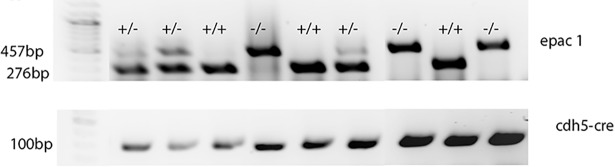
Genotyping of Epac1 floxed and Epac1 EC-KO mice. An agarose gel image from mouse ear punch samples from these mice shows effective knockout of Epac1 by Cdh5 Cre. Expected band sizes: Epac mutant 457bp, wildtype 276bp, Cdh5 Cre 300bp.

Epac1 is lost in the cdh5-Cre mice bred with Epac1 floxed mice. Fig 1 shows genotyping results for the mice used for the I/R experiments. We have previously reported that mice with this genotype also have loss of Epac1 in whole retinal lysates and in the retinal blood vessels [12].

**Fig 2:**
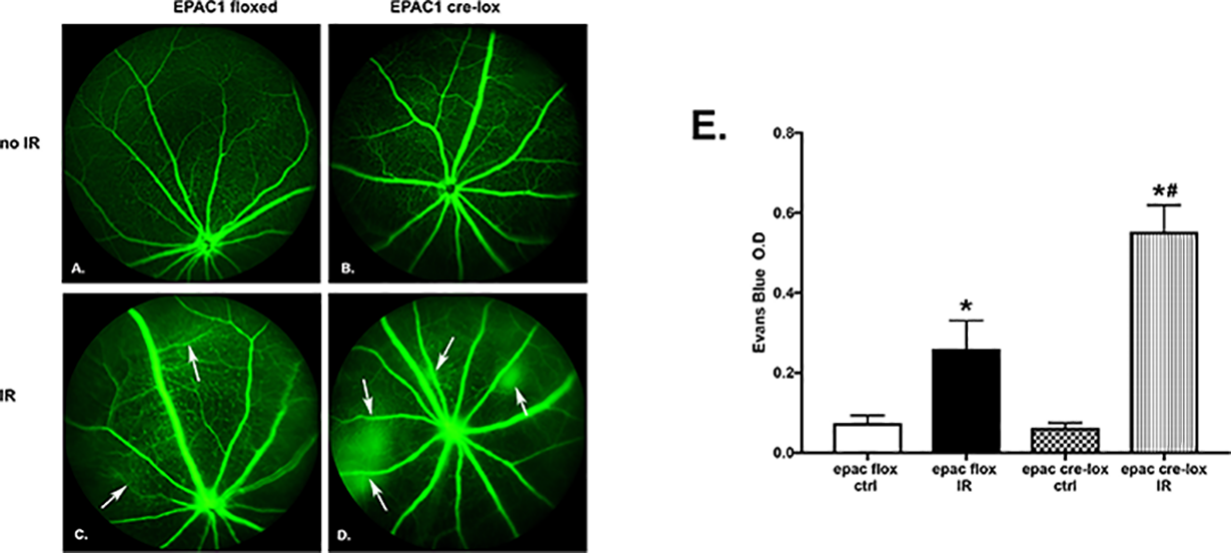
Fluorescein angiography of Epac1 floxed (left) and Epac1 endothelial cell specific knockout mice (right). Panel A and B are Epac1 floxed and Epac1 EC-KO without ischemia/reperfusion (I/R). Panel C and D are the respective groups of mice after exposure to I/R. Arrows point at spots of leakage. N = 4 for each group. Panel E is a quantification of leakage using the Evan’s Blue method. N = 4 for each group *P<0.05 vs. Epac1 floxed, #P<0.05 vs. Epac1 EC-KO.

Loss of Epac1 increased vascular leakage after I/R. To investigate the role of Epac1 in retinal vascular leakage in response to ischemia, we subjected Epac1 floxed and Epac1 endothelial cell specific knockout mice to I/R. Fig 2D shows that loss of Epac1 in endothelial cells led to increased vascular leaking after I/R compared to the Epac1 floxed mice (C). There was no difference in leakage without exposure to I/R (Fig 2A and 2B). In addition to fluorescein angiography, we also quantified retinal leakage using Evan’s Blue. Fig 2E shows that exposure to I/R increased leakage in the Epac1 floxed mice, but this leakage was significantly greater in the mice lacking Epac1.

**Fig 3:**
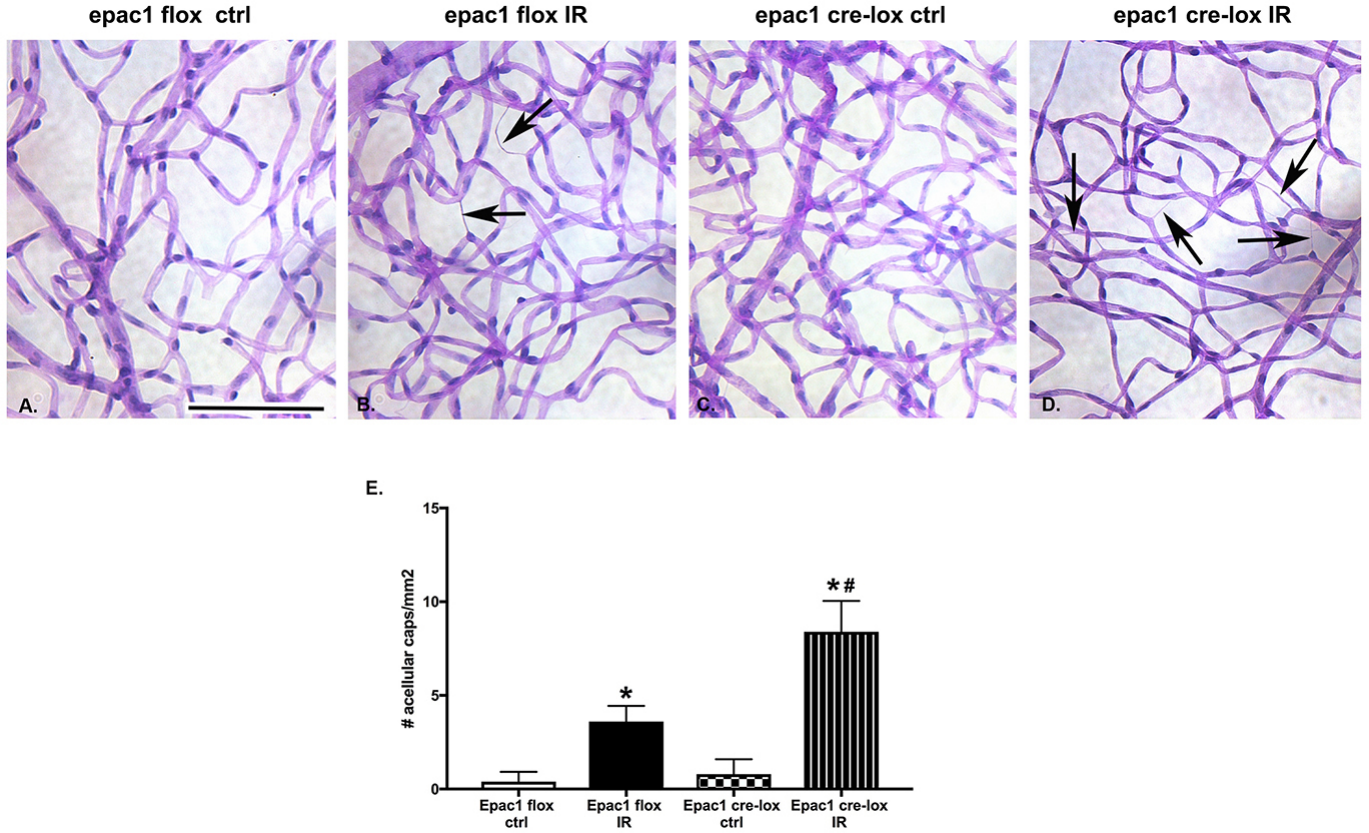
Measurement of degenerate capillaries in Epac1 floxed and Epac1 endothelial cell specific knockout mice. Panel A and C are Epac1 floxed and Epac1 EC-KO without ischemia/reperfusion (I/R). Panel B and D are the respective groups of mice after exposure to I/R. Panel E is quantification of the numbers of degenerate capillaries in all panels. Arrows point at degenerate capillaries. Scale bar is 20um. N = 4 for each group.

Epac1 decreases degenerate capillary formation after I/R. Ten days after exposure to I/R, we collected retinal samples from the Epac1 floxed and endothelial cell specific knockout mice and processed them for measurement of degenerate capillaries. Fig 3D shows that the numbers of degenerate capillaries is greater in the retinal flatmounts from the Epac1 EC-KO mice compared to the Epac1 floxed mice (Fig 3B). No differences were noted in retinas not exposed to I/R (Fig 3A and 3C). Panel E is quantitation of panels A-D showing significantly more degenerate capillaries in the Epac1 EC-KO mice.

**Fig 4:**
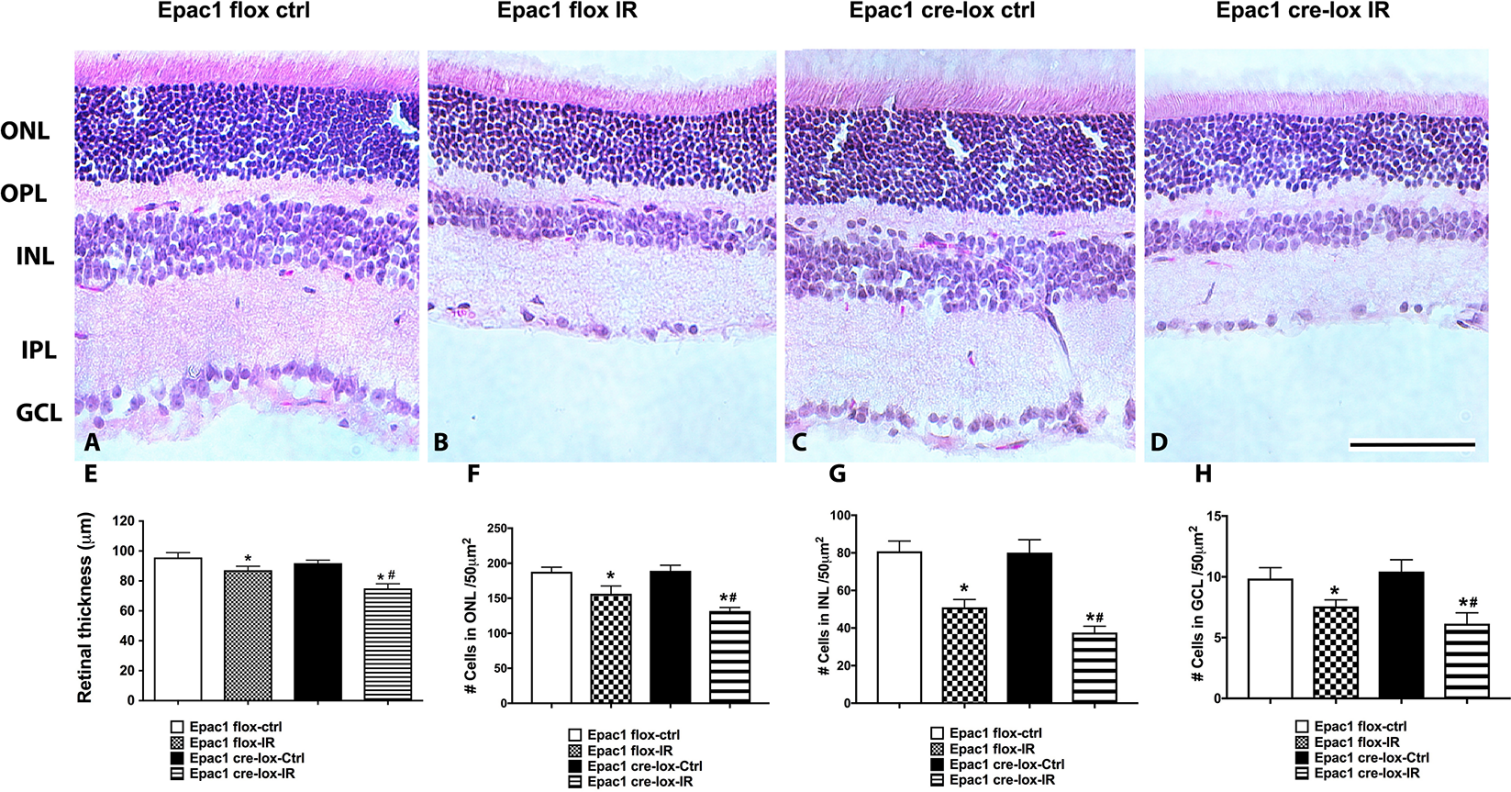
Measurement of retinal thickness and cell numbers in the ganglion cell layer (GCL) in Epac1 floxed and Epac1 endothelial cell specific knockout mice. Panel A and C are Epac1 floxed and Epac1 EC-KO without ischemia/reperfusion (I/R). Panel B and D are the respective groups of mice after exposure to I/R. Panel E is quantification of retinal thickness measurements from all panels, panel F is cell numbers in the outer nuclear layer, panel G is cell numbers in the inner nuclear layer, and panel H is cell numbers in the ganglion cell layer. Scale bar is 50um. *P<0.05 vs. Epac1 floxed, #P<0.05 vs. Epac1 EC-KO. N = 5 for each group.

Loss of Epac1 in the retinal vasculature reduces retinal thickness. While Epac1 is known to play a role in the vasculature [16], its role in the regulation of retinal neuronal changes is less clear. We used the Epac1 floxed and Epac1 EC-KO mice to investigate whether loss of Epac1 altered retinal thickness and cell numbers in the retina. Fig 4B and 4D shows a thinner retina when mice were subjected to the I/R protocol. No differences were noted in mice not exposed to I/R. Fig 4E is the quantification of total retinal thickness. The numbers of cells in specific layers are given in Fig 4F–4H. The data suggest that endothelial cell specific loss of Epac1 also causes neuronal damage after exposure to ischemia.

## Discussion

The goal of this work was to investigate the role of Epac1 on both vascular and neuronal retinal ischemic damage. It is becoming increasingly clear that ischemia can cause both neuronal and vascular damage to the retina [2, 17]. A number of studies have shown that key inflammatory pathways can induce retinal damage in response to ischemia, including TLR4 and HMGB1 [4, 5, 7]. We have previously reported that inhibition of HMGB1 by glycyrrhizin was effective in reducing retinal damage in response to I/R, with a focus on the retinal vasculature [8]. Our findings were in agreement with work in the brain exposed to I/R [18].

In addition to glycyrrhizin actions on HMGB1, we have also reported that Epac1 can reduce retinal inflammatory mediators [12], as well as the NLRP3 inflammasome [11]. Others have also reported that suppressing the NLRP3 inflammasome via pioglitazone ameliorated retinal damage after I/R [19]. Epac1 has been shown to reduce myocardial damage in response to I/R [20]. Additionally, work in muscle and endothelial cells showed that Epac1 reduced hyper-permeability following exposure to I/R [21]. We previously reported that Epac1 regulated ZO-1 and occludin in the mouse retina and in retinal endothelial cells [22]. We wanted to expand that work to measure whether Epac1 reduce retinal damage after exposure to I/R. We chose to focus on Epac1, as we reported that Epac1 regulated inflammatory mediators in retinal endothelial cells, while Epac2 did not have these actions [12]. Similar to our findings in the retina, work in a middle cerebral artery occlusion model showed that loss of Epac2 caused greater retinal swelling and oxidative stress [23]. Thus, our findings that loss of Epac1 caused exacerbated vascular leakage and neuronal and vascular damage in response to I/R agrees well with established literature in other targets and models.

In conclusion, Epac1 is protective against both retinal neuronal and vascular damage in response to I/R. Use of endothelial cell specific knockout mice also showed that Epac1 regulates retinal vascular leakage. Taken together, data suggest that maintenance of Epac1 levels in the ischemic retina is key to retinal health.
